# Biogenic selenium nanoparticles (SeNPs) from citrus fruit have anti-bacterial activities

**DOI:** 10.1038/s41598-021-84099-8

**Published:** 2021-02-26

**Authors:** Ghalia Batool Alvi, Muhammad Shahid Iqbal, Mazen Mohammed Saeed Ghaith, Abdul Haseeb, Bilal Ahmed, Muhammad Imran Qadir

**Affiliations:** 1 Institute of Molecular Biology and Biotechnology, Bahauddin Zakariya University, Multan, Pakistan; 2 Department of Clinical Pharmacy, College of Pharmacy, Prince Sattam Bin Abdulaziz University, Al-kharj, 11942 Saudi Arabia; 3 Department of Laboratory Medicine, Faculty of Applied Medical Sciences, Umm Al-Qura University, Makkah, Saudi Arabia; 4 Department of Clinical Pharmacy, College of Pharmacy, Umm Al-Qura University, Al-Abdia Campus, Taif Road, PO Box 13574, Makkah, 21955 Saudi Arabia; 5 School of Pharmacy, Nanjing Medical University, Nanjing, Jiangsu Province People’s Republic of China

**Keywords:** Nanobiotechnology, Nanoparticles

## Abstract

Nanotechnology deals with the synthesis of materials and particles at nanoscale with dimensions of 1–100 nm. Biological synthesis of nanoparticles, using microbes and plants, is the most proficient method in terms of ease of handling and reliability. Core objectives of this study were to synthesize metallic nanoparticles using selenium metal salt from citrus fruit extracts, their characterization and evaluation for antimicrobial activities against pathogenic microbes. In methodology, simple green method was implicated using sodium selenite salt solution and citrus fruit extracts of Grapefruit and Lemon as precursors for synthesizing nanoparticles. Brick red color of the solution indicated towards the synthesis of selenium nanoparticles (SeNPs). Nanoparticle’s initial characterization was done by UV–Vis Spectrophotometry and later FTIR analysis and DLS graphs via Zetasizer were obtained for the confirmation of different physical and chemical parameters of the nanoparticles. Different concentrations of SeNPs were used for antimicrobial testing against *E. coli*, *M. luteus*, *B. subtilis* and *K. pneumoniae* comparative with the standard antibiotic Ciprofloxacin. SeNPs possessed significant antimicrobial activities against all the bacterial pathogens used. Conclusively, SeNPs made from citrus fruits can act as potent antibacterial candidates.

## Introduction

From last few decades, Nanotechnology has become the most promising and advancing field because of its wide applications in applied sciences and technology^1^. In order to synthesize nanoscale materials, by using any biological source, nanotechnology is considered as emerging technical tool for their ecofriendly synthesis^2^. Nanoparticles display distinctive characteristics because of their high surface energy and large surface to volume ratio^3^. Metallic nanoparticles are very popular for their wide range of applications in different areas of science including physics, chemistry, material and biomedical sciences. They have numerous applications in optoelectronics, catalysis and diagnostic biological devices^2^. The size and properties of every nanoparticle vary depending upon the synthesis methods of nanoparticles and their source.

Synthesizing nanoparticles with different chemical composition, size and controlled mono-dispersity has become areas of research in nanotechnology^4^. Synthesis of nanoparticles can be carried out by various means for example physical, chemical and biological methods. Biological based synthesis using plants and their extracts, enzymes and microbes is considered as the most ecofriendly alternative to physical and chemical methods. Using plant-based synthesis methods are beneficial over other biological procedures as they eliminate the complex process of cell culture maintenance^5^.

Selenium is well known for its semiconductor and photoelectric properties. It also has great potential in the field different fields of science including medicine, biology, physics and chemistry. Selenium nanoparticles possess good adsorptive and biological activity because of interaction between nanoparticles and different functional groups (C–O, C–N. NH and COO–) of proteins^6^. These nanoparticles also exhibit antimicrobial, anticancer, antioxidant and enzyme inhibition activities but preparation of stable selenium nanoparticles is bit tricky^7^. Number of different plant extracts, microorganisms and enzymes has been utilized as a source for production of Selenium nanoparticles of variable size and morphology. Nano-sized Selenium particles are used for huge number of applications because of their advantageous features over bulk form for example low dosage, low toxicity and better reactivity^3^.

Citrus are the fruits belonging to genus. There are several instances of their reported antimicrobial activity^8^. Few other activities like antiviral, uricosuric, larvicidal, anti-hepatotoxic, anti-yeast and antimutagenic have been reported by citrus fruits. The antimicrobial activity of citrus has been found to be present due to a number of reasons like presence of volatile oily compounds, essential oils and various flavonoids. They have been widely employed for synthesizing metallic nanoparticles because of their notable activities. Citrus fruits have been selected as a source for Selenium nanoparticles synthesis because of their promising antibacterial properties^9^.

The objective of the current study was to synthesize selenium nanoparticles from citrus fruit i.e. *Citrus limon* (lemons) and *Citrus paradise* (grapefruits) extracts, their general characterization and their assessment for antibacterial activities against various pathogens.

## Results

**Figure 1:**
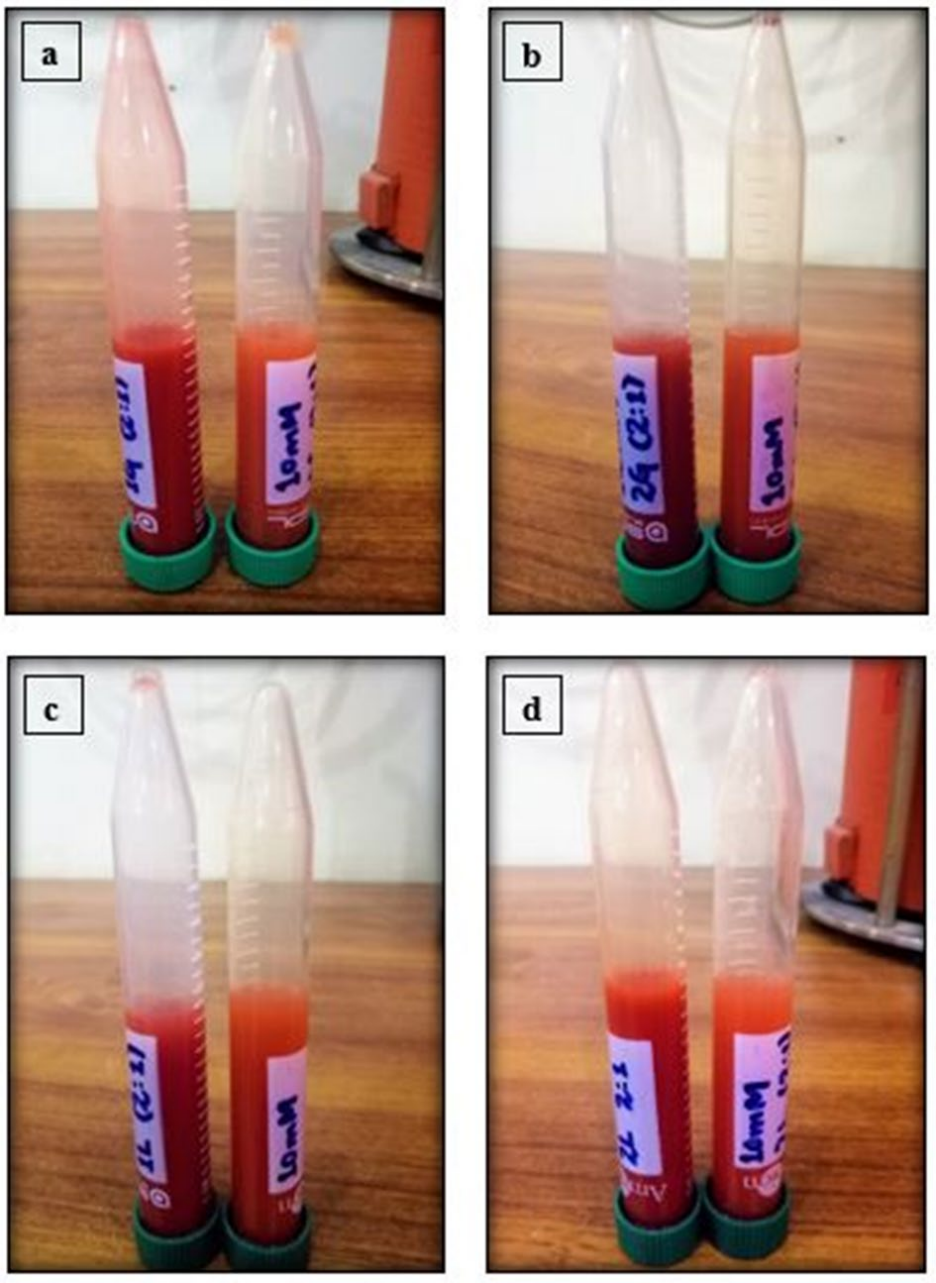
SeNPs synthesized from citrus fruit extracts; (**a**) from *Citrus paradisi* fresh juice extract, (**b**) from *Citrus limon*Fresh juice extract, (**c**) from *Citrus* paradisi fresh peels extract and (**d**) from *Citrus limon* fresh peel extract.

Selenium nanoparticles (SeNPs) synthesized from citrus fruits extracts via Biological method of nanoparticles synthesis are shown in Fig. 1.

**Figure 2:**
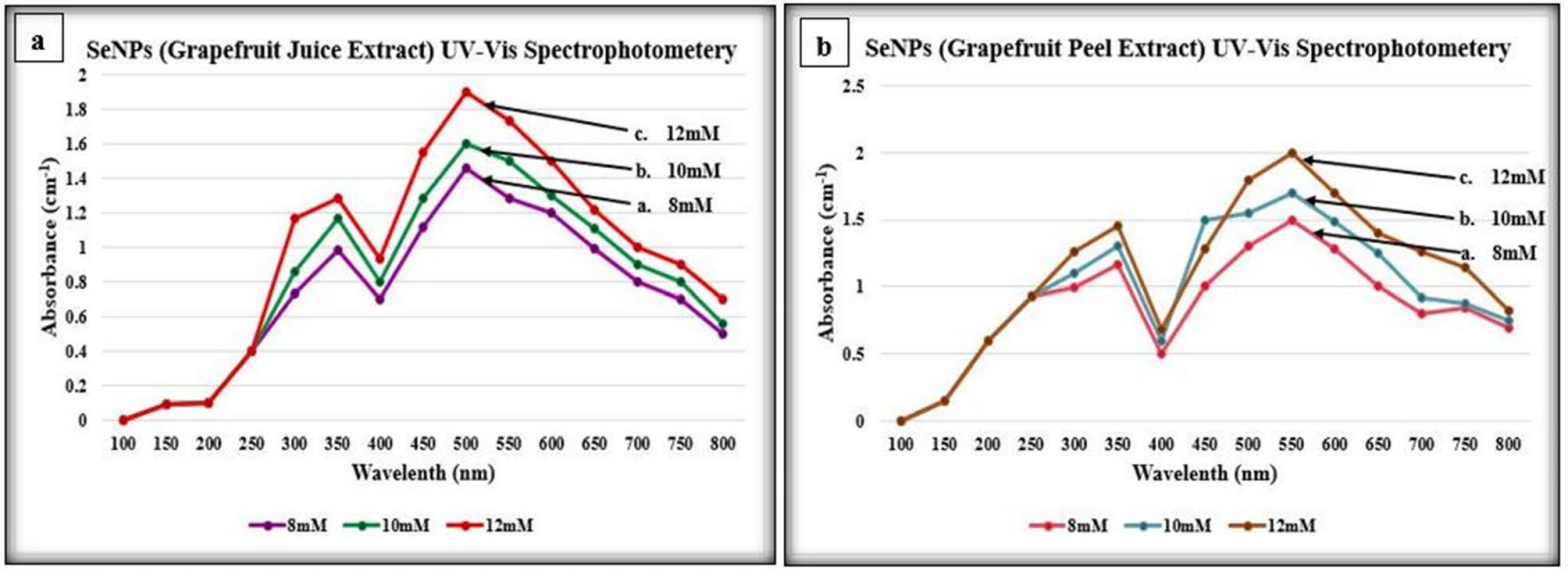
UV–Vis spectrophotometry analysis of SeNPs synthesized from *Citrus paradisi* (Grapefruit); (**a**) fresh juice extract, (**b**) fresh peels extract.

**Figure 3:**
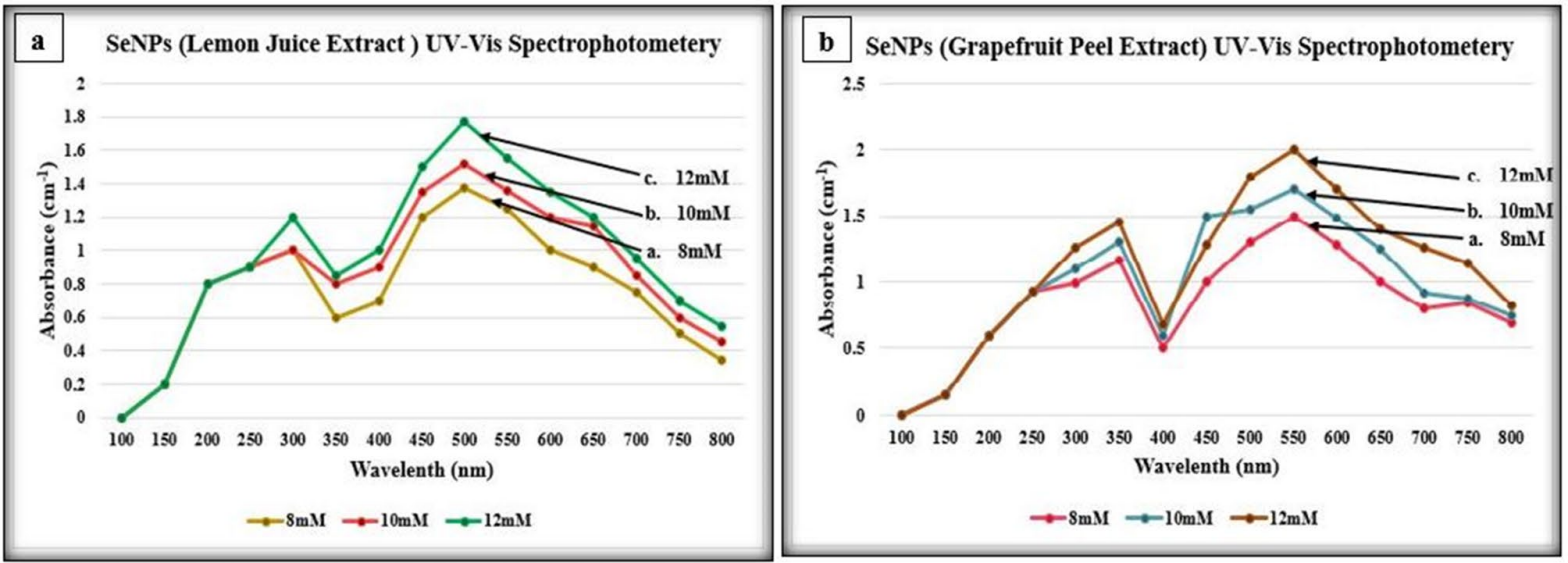
UV–Vis spectrophotometry analysis of SeNPs synthesized from *Citrus limon* (Lemon); (**a**) fresh juice extract, (**b**) fresh peels extract.

UV–Vis Spectrophotometry was performed for all the synthesized SeNPs from citrus fruits extracts and the synthesized SeNPs showed maximum absorption peaks between the range of 400–600 nm wavelength at absorbance of about 1.5–2. SeNPs synthesized from *Citrus paradisi* (Grapefruit) fresh juice extract showed the absorbance peaks at 350 nm and 500 nm while SeNPs synthesized from *Citrus paradisi* (Grapefruit) fresh peels extract displayed absorbance at 345 nm and 550 nm which is clearly demonstrated in the Fig. 2a,b respectively. In the same context, SeNPs that were synthesized using fresh juice extract of *Citrus limon* (Lemon) showed absorbance peaks at 300 nm and 500 nm whereas SeNPs synthesized from fresh peel extract of *Citrus limon* (Lemon) showed absorbance peaks at wavelengths of 350 nm and 550 nm clearly shown in Fig. 3a,b respectively.

**Figure 4:**
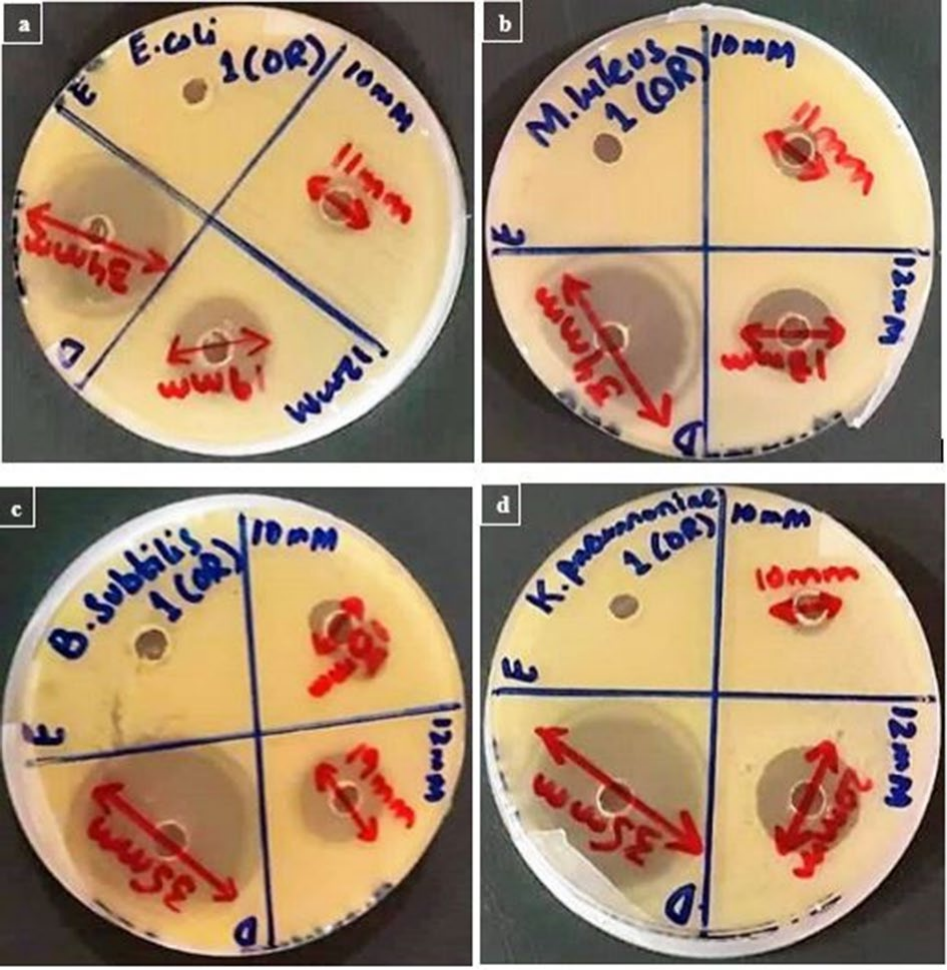
Antimicrobial activity analysis of SeNPs synthesized from *Citrus paradisi* (Grapefruit) fresh juice extract against bacterial pathogens; (**a**) *E. coli*, (**b**) *M. luteus*, (**c**) *B. subtilis*, (**d**) *K. pneumoniae*.

Antimicrobial activity of the synthesized SeNPs from different fruit extracts were evaluated through agar well diffusion method against bacterial pathogens comparative to the standard antibiotic Ciprofloxacin. The clear ZOIs of the synthesized SeNPs are shown in the figures and their values are described in the tables. ZOIs of SeNPs synthesized by *Citrus paradisi* fresh juice extract are displayed in Fig. 4.

**Figure 5:**
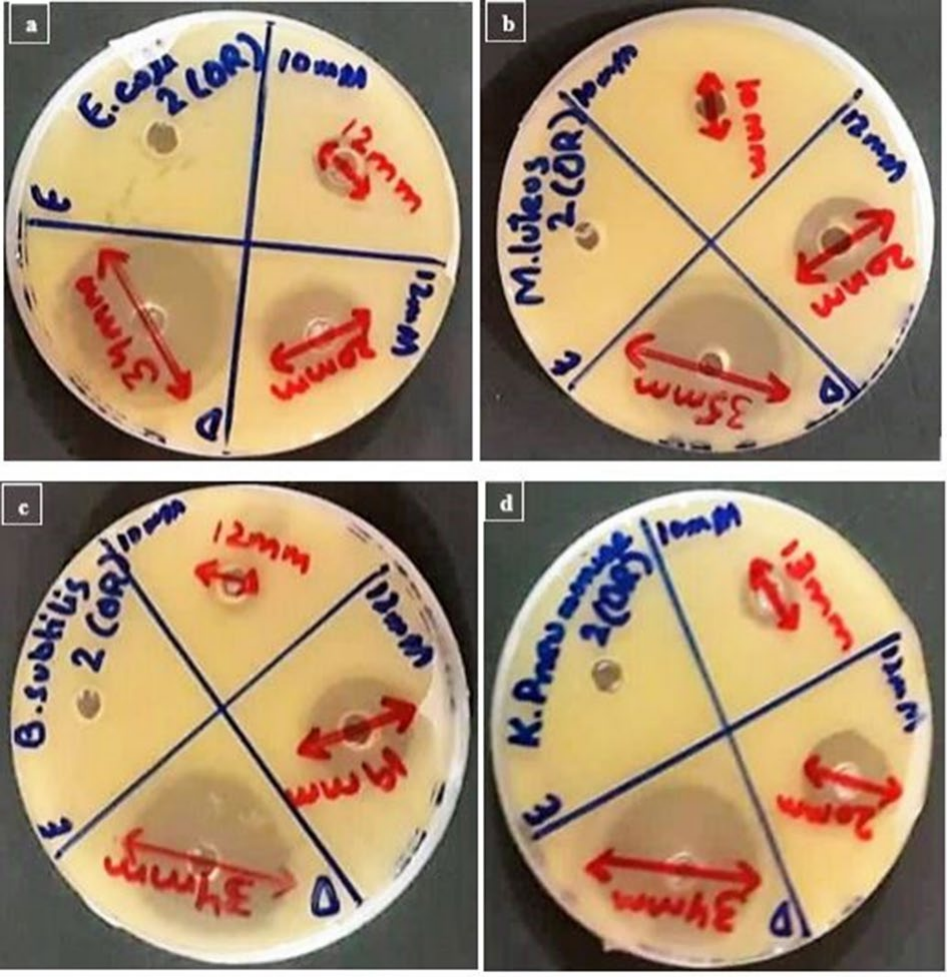
Antimicrobial activity analysis of SeNPs synthesized from *Citrus paradisi* (Grapefruit) fresh peels extract against bacterial pathogens; (**a**) *E. coli*, (**b**) *M. luteus*, (**c**) *B. subtilis*, (**d**) *K. pneumoniae*.

**Figure 6:**
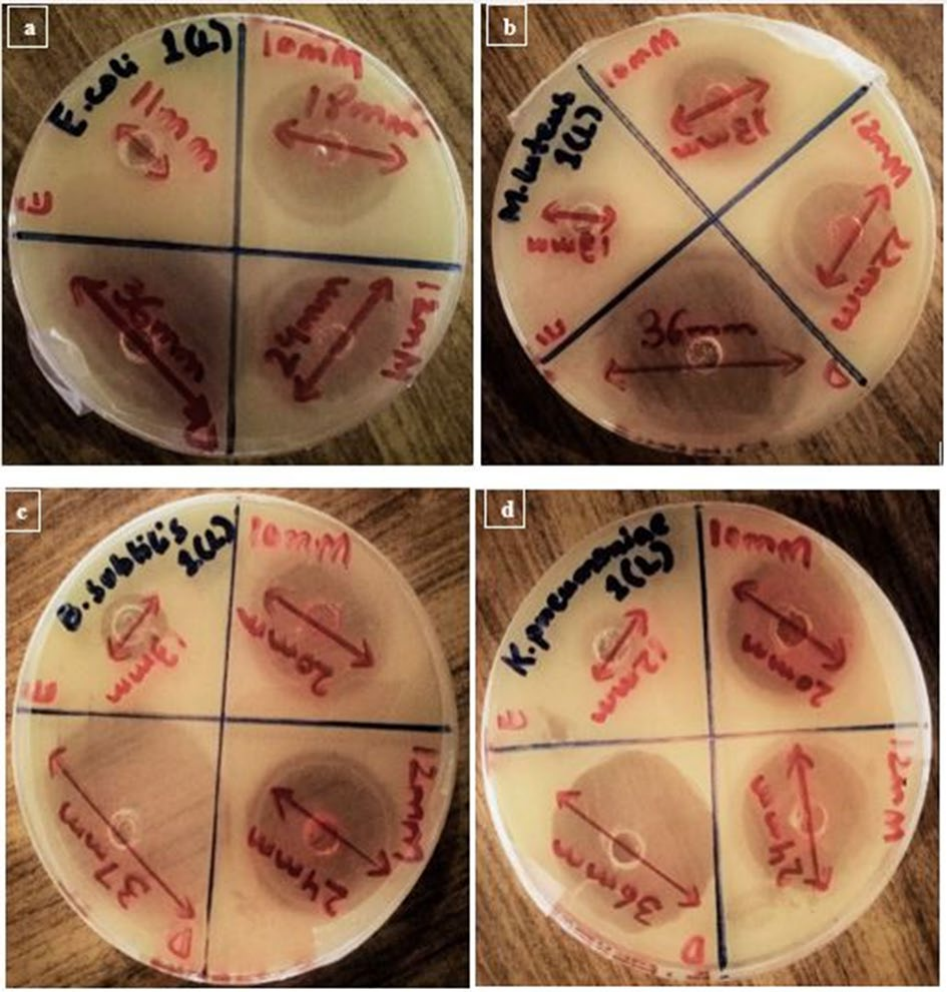
Antimicrobial activity analysis of SeNPs synthesized from *Citrus limon* (Lemon) fresh juice extract against bacterial pathogens; (**a**) *E. coli*, (**b**) *M. luteus*, (**c**) *B. subtilis*, (**d**) *K. pneumoniae*.

**Figure 7:**
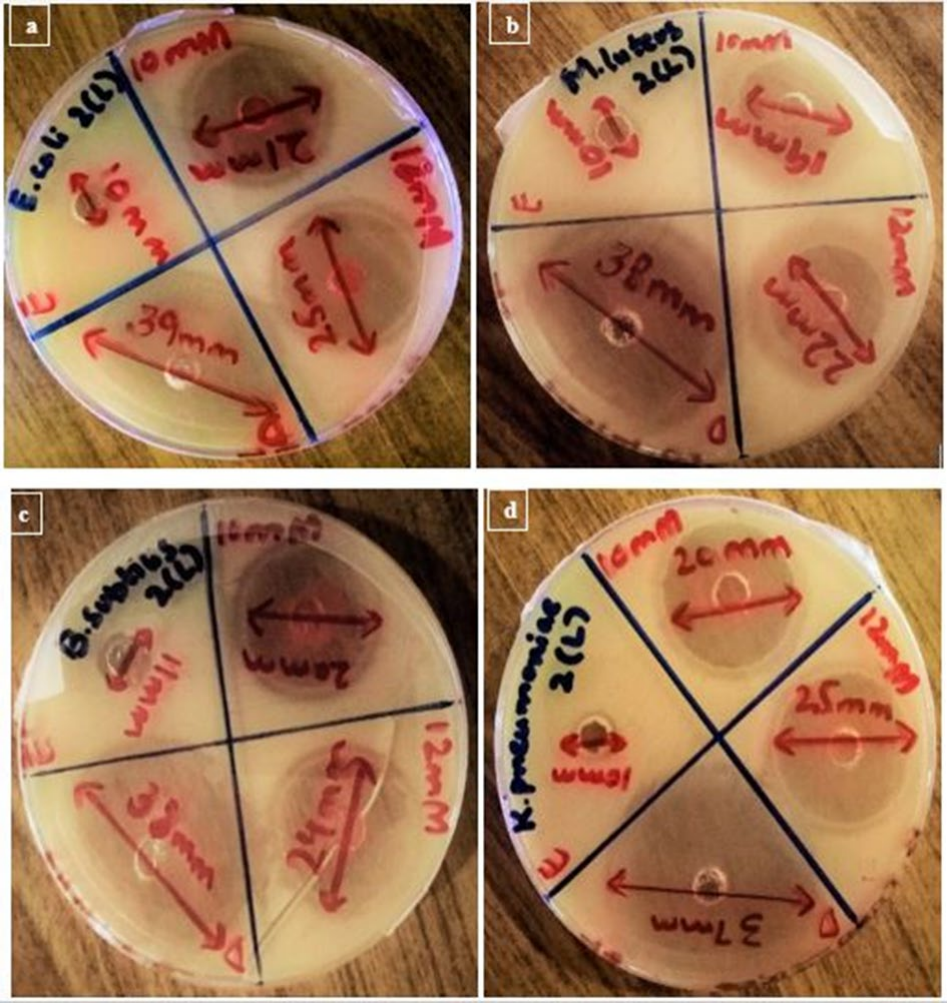
Antimicrobial activity analysis of SeNPs synthesized from *Citrus limon* (Lemon) fresh peels extract against bacterial pathogens; (**a**) *E. coli*, (**b**) *M. luteus*, (**c**) *B. subtilis*, (**d**) *K. pneumoniae.*

Antimicrobial activities of SeNPs synthesized from the Citrus fruit are given in Tables 1, 2, 3 and 4. Figures 5, 6 and 7 are the representation of Zone of inhibitions (ZOIs) of the synthesized SeNPs. SeNPs synthesized from Lemon extracts with 12 mM Sodium selenite concentration exhibits highest antimicrobial activity against bacterial pathogens specifically against *E. coli* and *K. pneumoniae* with ZOI of 25 mm. So, further characterization tests were performed for that sample of SeNPs. Results were statistically analyzed. The mean value and the standard deviation of all the replicates were calculated. The nanoparticles synthesized from both the citrus fruits were compared for their antibacterial activity. The resultant (p value less than 0.05) of t-test to compared two citrus fruits for their activity (*Citrus limon* and *Citrus paradisi*) have statistical differences. *Citrus limon* has a better activity. The p-value < 0.05 was considered as significant. The results confirmed that SeNPs synthesized from *Citrus limon* (Lemon) fresh peels extract showed antimicrobial activity comparable with Standard (Ciprofloxacin) with p < 0.05.

**Table 1:** Zone of inhibition (mm) of SeNPs synthesized from *Citrus paradisi* (Grapefruit) fresh juice extract in comparison with the standard drug and control extract against bacterial pathogens.

Standard and sample	Replicates	*E. coli*	*M. luteus*	*B. subtilis*	*K. pneumoniae*
Standard drug	R1	34.6	33.9	35	33.9
	R2	34.8	34.2	35.2	34.8
	R3	32.6	33.9	34.8	36.3
	Mean ± SD	34 ± 1.216	34 ± 0.173	35 ± 0.200	35 ± 1.212
10 mM	R1	11.6	11.8	14.6	10.4
	R2	10.2	10.2	12.4	8.9
	R3	11.2	11	12	10.7
	Mean ± SD	11 ± 0.721	11 ± 0.800	13 ± 1.400	10 ± 0.964
12 mM	R1	20.2	17.7	20	21.9
	R2	19	19.3	19.6	19.1
	R3	17.8	17	17.4	19
	Mean ± SD	19 ± 1.200	18 ± 1.178	19 ± 1.400	20 ± 1.646

p-value < 0.05.

**Table 2:** Zone of inhibition (mm) of SeNPs synthesized from *Citrus paradisi* (Grapefruit) fresh peels extract in comparison with the standard drug and control extract against bacterial pathogens.

Standard and sample	Replicates	*E. coli*	*M. luteus*	*B. subtilis*	*K. pneumoniae*
Standard drug	R1	34.8	36.1	33.6	33.8
	R2	33.9	35.8	34.2	35.1
	R3	33.3	33.1	34.2	33.1
	Mean ± SD	34 ± 0.754	35 ± 1.652	34 ± 0.346	34 ± 1.014
10 mM	R1	13.5	9.9	12.4	13
	R2	12	10.1	10.8	12.7
	R3	10.5	10	12.8	13.3
	Mean ± SD	12 ± 1.500	10 ± 0.100	12 ± 1.058	13 ± 0.300
12 mM	R1	21.4	19.3	18.8	20
	R2	20.1	20.2	19.2	21.6
	R3	18.5	20.5	19	18.4
	Mean ± SD	20 ± 1.452	20 ± 0.624	19 ± 0.200	20 ± 1.600

p-value < 0.05.

**Table 3:** Zone of inhibition (mm) of SeNPs synthesized from *Citrus limon* (Lemon) fresh juice extract in comparison with the standard drug and control extract against bacterial pathogens.

Standard and sample	Replicates	*E. coli*	*M. luteus*	*B. subtilis*	*K. pneumoniae*
Standard drug	R1	36.4	35.8	37.3	35.6
	R2	35.9	36.2	37.7	36.3
	R3	35.7	36	36	36.1
	Mean ± SD	36 ± 0.360	36 ± 0.200	37 ± 0.888	36 ± 0.360
10 mM	R1	18	18.5	21.3	20
	R2	17.4	19	20	19.2
	R3	18.6	16.5	18.7	20.8
	Mean ± SD	18 ± 0.600	18 ± 1.322	20 ± 1.300	20 ± 0.800
12 mM	R1	25	21.6	24.2	25.2
	R2	23.9	22	23.6	24
	R3	23.1	22.4	24.2	22.8
	Mean ± SD	24 ± 0.953	22 ± 0.400	24 ± 0.346	24 ± 1.200

p-value < 0.05.

**Table 4:** Zone of inhibition (mm) of SeNPs synthesized from *Citrus limon* (Lemon) fresh peels extract in comparison with the standard drug and control extract against bacterial pathogens.

Standard and sample	Replicates	*E. coli*	*M. luteus*	*B. subtilis*	*K. pneumoniae*
Standard drug	R1	39	37.6	38	36.2
	R2	38.5	37.4	38.5	37.3
	R3	39.5	39	37.5	37.5
	Mean ± SD	39 ± 0.500	38 ± 0.871	38 ± 0.500	37 ± 0.700
10 mM	R1	21.3	19.1	21	18.4
	R2	22.4	19.8	20.2	19.8
	R3	19.3	18.1	18.8	21.8
	Mean ± SD	21 ± 1.571	19 ± 0.854	20 ± 1.113	20 ± 1.708
12 mM	R1	26.2	21.7	24.2	26
	R2	25.4	22	24.6	25.8
	R3	23.4	22.3	23.2	23.2
	Mean ± SD	25 ± 1.442*	22 ± 0.300	24 ± 0.721*	25 ± 1.562*

*p-value < 0.05.

**Figure 8:**
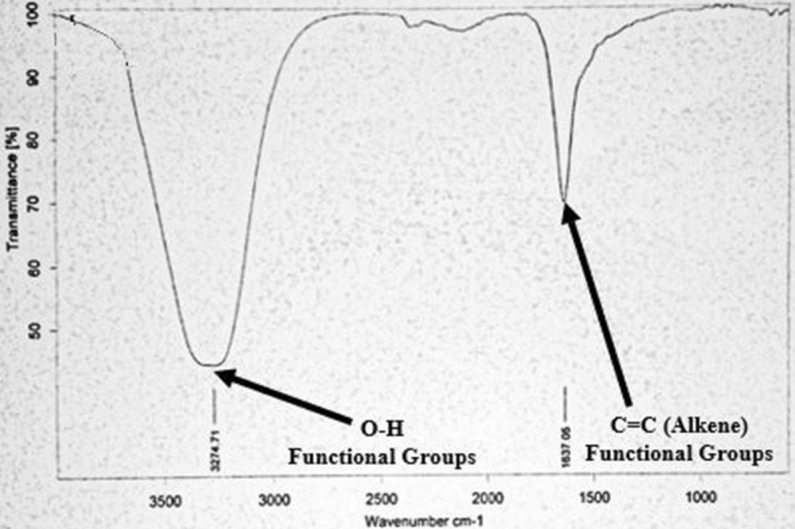
FTIR band spectrum study of SeNPs synthesized from *Citrus limon* (Lemon) fresh peels extract.

Fourier Transform Infra-Red (FTIR) Spectroscopy was performed using FTIR spectrometer for the detection and presence of functional groups involved in the selenium nanoparticle synthesis. Wavelength absorbance reading of Infra-red radiations was checked [for SeNPs synthesized from *Citrus limon* (Lemon) peels extract] between the range of 1000–3500 cm^−1^. Figure 8 displayed the two absorbance peaks for SeNPs synthesized from *Citrus limon* (Lemon) fresh peels extract at 3274.74 cm^−1^ and 1637.05 cm^−1^. The peak between 3200 and 3300 cm^−1^ confirmed the presence of O–H bonded stretching and strong vibrations due to presence of alcohol and phenol functional groups. N–H stretching of Amide A in proteins was also detected in that region. Similarly, for the peak in the region of 1600–1700 cm^−1^, C=C stretching, C-N stretching and Amides functional groups of amides I & II with C–H bonds with the presence of alkenes.

**Figure 9:**
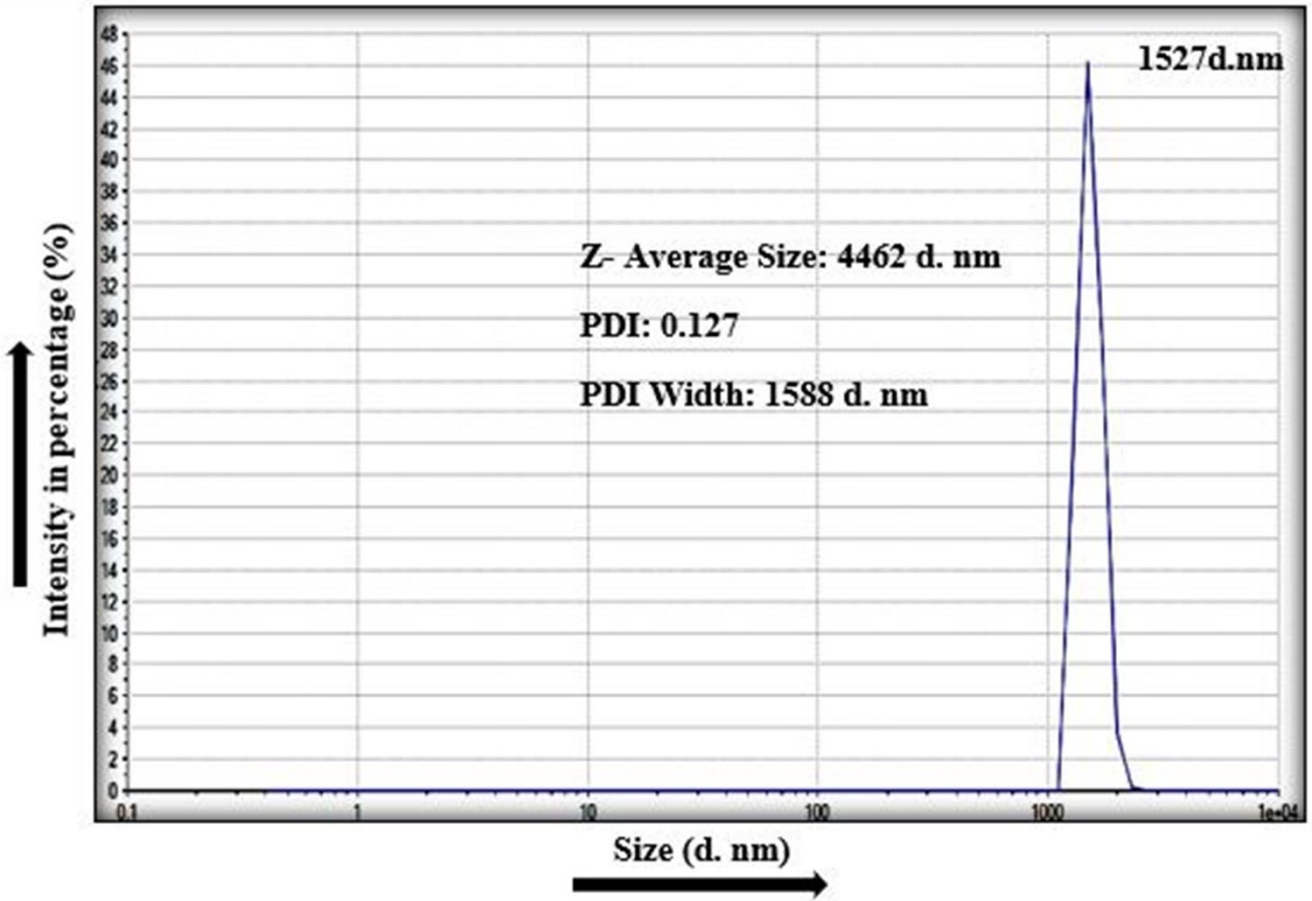
Dynamic light scattering study of SeNPs synthesized from *Citrus limon* (Lemon) fresh peels extract.

Zetasizer was used for the determination of size of the synthesized SeNPs exhibiting highest antimicrobial activity against all the bacterial pathogens i.e. SeNPs synthesized from Lemon peel extract, using Dynamic light scattering (DLS) phenomena. DLS measures the hydrodynamic volume of the particles. Figure 9 represents the Dynamic Light Scattering Analysis (DLS) through Zetasizer. On the x-axis there was hydrodynamic diameter the SeNPs as diameter in nanometer (d.nm), while on y-axis values for scattering intensity from the particles were plotted. The test sample of the SeNPs synthesized from *Citrus limon* (Lemon) Peels extract was analyzed for measuring its size through dynamic light scattering phenomena. A graph was obtained with one visible peak within a range of 1100–3500 nm (diameter). The maximum peak was observed at 1527 d.nm/100.0%. The average Zeta size of the synthesized SeNPs was about 4462 d.nm and its PDI (polydispersion index) value was 0.127 with PDI width of 1588 d. nm.

## Discussion

The basic objective of this study was the biogenic synthesis of SeNPs from two citrus species, i.e., *Citrus paradisi* and *Citrus limon* and their further analysis as potential antibacterial agents. The primary reason for selecting two citrus species is to find out which performs best against bacterial species. Naturally citrus fruits are known to have several nutritional compounds. Other than these beneficial phytochemicals and phenolic compounds, they are known to possess several biological properties^10^. In the present era, antimicrobial infections are the most common threat to human health. The use of antibiotics can cure bacterial infections, but their frequent use makes bacteria resistant against such antibiotics. The need of developing new antibacterial drugs is the need of present time. Selenium also possesses several biomedical applications. Selenium nanoparticles synthesized from citrus fruits can amplify the biomedical properties of both selenium and citrus fruits.

Several researchers have synthesized selenium nanoparticles using leaves of citrus plants, but we have adopted the biological method for synthesizing SeNPs from *Citrus paradisi* and *Citrus limon*^3^. The bacterial strains against which antibacterial property of the nanoparticles were tested can pose a major threat to human health. In this research, SeNPs were synthesized using fresh juice and peel extracts of grapefruit and lemon. Two parts of the fruit extract and one part of the salt solution was mixed. The solution after incubation for the optimum time and temperature and after a visible color change from slight yellowish to bright brick red color confirmed the reduction of sodium selenite into SeNPs. A visible color change from light yellow to brick red color indicated the synthesis of SeNPs. Vyas and Rana^4^ synthesized SeNPs using *Allium sativum* extract. A similar color change from yellow to brick red was observed which confirmed the synthesis of selenium nanoparticles.

The synthesized SeNPs were then used for UV–Vis Spectrophotometer Analysis for the confirmation of the reduction of sodium selenite into SeNPs. The synthesized SeNPs from *Citrus paradisi* and *Citrus limon* showed absorbance peaks between a range of 300–550 nm. In a similar study of SeNP synthesis from *Citrus limon* leaves by KS Prasad and colleagues, the UV–Vis spectrophotometer analysis of the synthesized SeNPs was done between 250–750 nm. The synthesized SeNPs exhibited absorbance peak at 395 nm^3^.

Then Functional groups involved in the reduction of sodium selenite into SeNPs were evaluated via FTIR spectroscopy of all the synthesized SeNPs. Absorbance reading of Infra-red radiations was checked for each sample between the ranges of 1000–3500 cm^−1^. All the samples of synthesized SeNPs in this study exhibited peaks between two ranges, i.e. 1600–1700 cm^−1^ and 3200–3300 cm^−1^. The absorbance peaks between 3200 and 3300 cm^−1^ confirmed the presence of O–H bonded stretching and strong vibrations due to presence of alcohol and phenol functional groups. N–H stretching of Amide A in proteins was also detected in that region. Similarly, for the peaks in the region of 1600–1700 cm^−1^, C=C stretching, C–N stretching and Amides functional groups of amides I & II with C-H bonds with the presence of alkenes. Borna Fardsadegh and Hoda Jafarizadeh-Malmiri synthesized SeNPs from aloe vera leaf extract. The FTIR spectroscopy showed that the synthesized nanoparticles exhibited peaks at 1635.52 cm^−1^, and 3454.3 cm^−1^. The peak in region between 1600–1700 cm^−1^ confirmed the presence of amide group while the peak between 2900 and 3200 cm^−1^ represented the O–H vibrations^11^.

In order to cope with the serious bacterial infections, the synthesized SeNPs were analyzed for antibacterial activity against harmful pathogens. The pathogens against which the synthesized SeNPs of this study tested were: *E. coli, M. luteus, B. subtilis* and *K. pneumoniae*. All these bacteria possess a serious threat to human health. Our synthesized SeNPs showed ZOI from 18 to 25 mm. All the nanoparticle concentrations were tested in comparison with the standard antibiotic drug ciprofloxacin. The maximum ZOI was showed by 12 mM concentration SeNPs from fresh lemon peel extract against *E. coli* and *K. pneumoniae*. The maximum zone is 25 mm. Many researchers have synthesized selenium nanoparticles but the antimicrobial activity of SeNPs from lemon (fresh juice & fresh peel) and grapefruit (fresh juice & fresh peel) against harmful bacterial pathogens in this manuscript correspond to a novel research. This research also supported the fact that SeNPs synthesized from citrus fruits especially lemon can act as potential antibacterial agents in the coming future.

SeNPs which showed highest antimicrobial activity, i.e. SeNPs synthesized from *Citrus limon* (Lemon) fresh peels extract were further used for the size determination via Zetasizer and Dynamic Light Scattering (DLS). A graph was obtained with one visible peak within a range of 1100–3500 d.nm. The maximum peak was observed at 1527 d.nm/100.0%. The size measure through dynamic light scattering is actually a bit larger than the actual size of the nanoparticles. The reason for the large measured size of the nanoparticles is due to the fact that DLS measures the hydrodynamic volume of the particle in aqueous state^12^. Sometime the particles in the liquid medium form aggregates. That’s why DLS phenomena measures the size of the aggregates rather than measuring the size of single nanoparticle^13^. Generally, PDI value ranges from 0 to 1. The particles with PDI from 0 to 0.08 are monodisperse sized particles, 0.08–0.7 is the middle range of particle size while 0.7–1 PDI value shows the polydispersity of the particle sizes. The particle synthesized in this study exhibited PDI value in range of 0.08–0.7 and PDI value under 0.3 is considered as the accepted or prioritized value in case of pharmaceutical NPs. Garima Sharma and colleagues synthesized SeNPs from dried *Vitis vinifera* (raisin) extract and used the DLS technique to measure the size of the synthesized nanoparticles^12^.

## Conclusion

Nanotechnology is the branch of science which deals with the dimensions in range of nanoscale. Nanoparticles can be produced from ceramics, polymers, metals etc. There are different types of metallic nanoparticles including Gold, Silver, Copper, Iron, Selenium and many others. Biological synthesis of SeNP can be done by bacteria, fungi, and plants (Green Synthesis). Green synthesis usually involves the amalgamation of Selenium salt solution with plant extracts to produce Nanoparticles. In this study, the antimicrobial activity of Citrus fruits extracts based SeNPs was analyzed. *Citrus limon* and *Citrus paradisi* were used as green source*.* Green synthesis methodology was used in this synthesis, and the color change to brick red was selected as an initial indicator of SeNPs synthesis. The confirmation and characterization tests of these SeNPs were performed. These tests involved UV Visible Spectrophotometer analysis, FTIR spectroscopy and Zetasizer analysis through Dynamic Light Scattering (DLS). After the confirmation of SeNPs synthesis, antimicrobial activities were checked against four bacterial cultures, *E. coli, K. pneumoniae, M. leteus,* and *B. subtilis.* The activity of SeNPs was compared with a standard antibiotic (Ciprofloxacin). The results showed that selenium nanoparticles synthesized from citrus fruits (*Citrus paradisi* and *Citrus limon*) possess notable antibacterial activities. They can act as a potential candidate for making promising antibacterial drugs as a replacement of antibiotics.

## Materials and methods

### Sample collection

Citrus fruits i.e. *Citrus limon* (lemons) and *Citrus paradise* (grapefruits) were purchased from Local fruit market of Pakistan while metal salt; Sodium selenite (Na_2_SeO_3_) was purchased from Sigma Aldrich distributions.

### Biosynthesis of selenium nanoparticles (SeNPs)

#### Fruit and peel extract preparation

Fruits samples were thoroughly washed with distilled and double distilled water to remove impurities and dust particles followed by drying of fruits with sterilized paper towels. Fruits were cut down using sterilized knife and fresh juice extracts of *Citrus limon* and *Citrus paradisi* were prepared by directly squeezing the juice out of fruit and then it was filtered through muslin cloth^14^ followed by centrifugation at 5000 rpm for 20 min at RT. The resultant supernatant was separated from the pellet in labelled sterilized glass media bottles and stored at 4 ℃ for further use extracts^5,15,16^. In the same context fresh peel extracts were prepared firstly by peeling the citrus fruits and then using sterilized knife, their peels were cut down into small pieces. About 70 g of the fresh peels were added in 300 mL of the double distilled water and boiled for 10–20 min in a water bath^9,17^. After boiling the peel extract was filtered through a series of Whatman filter papers with different pore sizes and the resultant filtrate was stored at 4℃ in labelled sterilized glass media bottles^18–20^.

#### Sodium selenite (Na_2_SeO_3_) salt solution preparation

For preparing sodium selenite solution, firstly a 1 M stock solution was prepared in 50 mL and from that stock solution further dilutions of 8 mM, 10 mM and 12 mM were prepared which were used for nanoparticle synthesis.

#### Synthesis of selenium nanoparticles (SeNPs)

Sodium Selenite (Na_2_SeO_3_) solution of 8 mM, 10 mM and 12 mM were mixed with fresh juice extracts and fresh peel extracts of *Citrus paradisi* and *Citrus limon*in a ratio of 2:1 (2-parts fruit extracts and 1-part sodium selenite solution) in separate beakers. The mixtures were then stirred on magnetic stirrer for 25–30 min to obtain a homogenous mixture. The pH was adjusted at 7 i.e., neutral. It was then subjected to orbital shaker at 70 ℃ in dark condition for 3 h at 200 rpm. After 3 h a slight color change was observed in all the sample mixtures and after 3 h the temperature was reduced to 37 ℃ for 72 h. After 3 days a strong color change was observed from pale yellow to brick red in all solutions. The sample tubes were then replaced from shaker to the incubator with same temperature condition. After further 2–3 days of incubation period the nanoparticles settled down at the base of the falcon tube^12,21,22^.

### Antimicrobial activity analysis

#### Bacterial sample collection and inoculum preparation

Bacterial Samples were provided by the Institute of Molecular Biology and Biotechnology (IMBB), Bahauddin Zakariya University (BZU), Multan, Pakistan. Four Bacterial cultures, *Escherichia coli* (*E. coli*)*, Micrococcus luteus* (*M. luteus*)*, Klebsiella pneumoniae* (*K. pneumoniae*) and *Bacillus subtilis* (*B. subtilis*) were used to test the antibacterial activity of synthesized SeNPs. For bacterial inoculum preparation, nutrient broth was prepared and with the aid of sterilized wire loop, bacterial culture was taken and mixed in the sterilized nutrient broth. The inoculum was used after incubation of 24 h at 37 ℃.

#### Agar well diffusion assay

Agar well diffusion assay was used for the evaluation of antimicrobial activity of synthesized SeNPs against bacterial pathogens. A 6 mm metallic borer was used for making wells. Ciprofloxacin was used as a standard drug for the assessment of antimicrobial activity.

### Characterization of selenium nanoparticles (SeNPs)

#### UV–vis spectrophotometer

A small aliquot of about 1 mL of the SeNPs of all the three concentrations i.e. 8 mM, 10 mM and 12 mM was used for UV–Visible spectrophotometer analysis after intervals of 3 h, 24 h and 72 h in order to observe the reduction of Se ions in the reaction mixture. The maximum absorption at the relevant wavelength was noted. The wavelength was set between 100 and 800 nm (nm) and the desired range to observe the peak for SeNPs was 300–800 nm. Double distilled water was used as a blank control in that analysis^4,23–28^. The spectrophotometer used for measuring the wavelengths of the SeNPs was PG instrument-t80 UV/Vis Spectrophotometer.

#### Sample preparation for further characterization

For sample preparation for further characterization tests of SeNPs, the nanoparticle mixture was subjected to repeat centrifugation for 3–4 times at 15,000 rpm for 20 min. after every centrifugation the supernatant was discarded and replaced with deionized water. After final centrifugation, the sample tubes were placed upside down on a sterilized paper towel in order to remove any extra water inside the tube. After 5–10 min the nanoparticles were oven dried and solid nanoparticles were obtained which were further used for Characterization tests^3,4,27^.

#### Fourier transform infra-red (FTIR) spectroscopy analysis

Fourier Transform Infra-Red (FTIR) Spectroscopic studies were done using Bruker Germany Alpha FTIR spectrophotometer of Bahauddin Zakariya University, Multan, Pakistan^27^. FTIR analyses were done for the determination of the functional groups present at the surface of the nanoparticles that might be involved in the sorption process. The dried nanoparticle samples were diluted in potassium bromide (KBr) solution. FTIR scan analysis range for the detection of the samples at peaks were between 1000 and 3500 cm^−1^^4,23,25,26,29,30^.

#### Dynamic light scattering (DLS)

For determining the size and morphological characteristics of the SeNPs, Zetasizer was employed. Zetasizer is used for the determination of three main properties of nanomaterials like its zeta potential, molecular weight and its size. The method by which the size of the nanoparticle is determined in Zetasizer is known as Dynamic Light Scattering (DLS). This procedure was carried out at 25℃ using Zetasizer Nano S90. Those nanoparticles which showed maximum antimicrobial activity were used to measure the size via Zetasizer^31,32^.

### Statistical analysis

The true experimental research design was used for performing the experiments. SPSS ver. 22 was used for analyzing the data. p < 0.05 was considered as significant.

### Ethical approval

Experimental organisms were not used.

## Data Availability

All the other data that support the findings of this study are available from the corresponding author upon request.
